# Role of Fetal Stem Cells in Maternal Tissue Regeneration

**Published:** 2007-09-06

**Authors:** Jiang F. Zhong, Leslie P. Weiner

**Affiliations:** 1 The Department of Neurology, Keck School of Medicine, University of Southern California, Los Angeles, CA, 90033, U.S.A; 2 Department of Pathology, Keck School of Medicine, University of Southern California, Los Angeles, CA, 90033, U.S.A

**Keywords:** Fetal stem cells, Microchimerism, Injury introduction

## Abstract

Microchimerism refers to the status of harboring cells from another individual at low levels. It is well known that cells traffic bidirectionally between fetus and mother during pregnancy. This situation resembles a naturally occurring long lasting fetal stem cell transplantation. The fetus acts as the donor and the mother acts as the recipient. To study the role of microchimerism in tissue regeneration, we constructed a murine microchimerism model with wild type C57BL/6J female mice carrying progenies which expressed green fluorescent proteins (GFP). Our data indicated that skin injuries in the female mice during pregnancy increased microchimerism of GFP expressing cells from the GFP transgenic progenies. The GFP positive cells also appeared at the site of spinal cord where injury occurred during pregnancy. Our study suggests that the amount of fetal cells in maternal mice significantly increased if injuries occurred during pregnancy. Fetal stem cells appear to respond to maternal injury signals and may play a role in maternal tissue regeneration during pregnancy.

## Introduction

Microchimerism refers to the status of harboring cells from another individual at low levels. It is well known that cells traffic bidirectionally between fetus and mothers during pregnancy in human (Bianchi et al. 1996; Nelson et al. 1998; Maloney et al. 1999; Lo et al. 2000; O’Donoghue et al. 2003; Khosrotehrani et al. 2006) and mouse.(Liegeois et al. 1981; Philip et al. 1982) It has been reported that fetal DNA presented in women for as long as 27 years following the birth of their last child, and maternal cells presented in adult offspring up to 28 years of ages.(Bianchi et al. 1996; Bianchi 1998; Maloney et al. 1999) The number of fetal cells in maternal circulation between 18 and 22 weeks of pregnancy was estimated to be approximately 2–6 cells/ml.(Krabchi et al. 2001) Because the number of fetal cells is small (hence called microchimerism), the role of these fetal cells in tissue regeneration has not been fully appreciated. Many studies of microchimerism focus on its effects on the host immune system, and its role in autoimmune diseases.(Nelson et al. 1998; Nelson 1999; Khosrotehrani et al. 2003; Lambert et al. 2003; Adams et al. 2004; Khosrotehrani et al. 2006) Recent studies suggest that these fetal cells have stem cell-like properties (Khosrotehrani et al. 2004; Khosrotehrani et al. 2005; Tan et al. 2005).

Using a congenic murine model, we showed here that the fetal cells in the pregnant mice are significantly increasing at the injury sites if injuries occurred during pregnancy. Our data also indicated that fetal cells participated in the repair of skin injury and in spinal cord injury. The fetal cell regenerated tissues suggest that the increase of microchimerism is due to the proliferation of fetal cells rather than migration of fetal cells. Understanding this repairing mechanism is important in clinical application of fetal stem cells for regenerative medicine. The murine injury model reported here provides a platform to study the role of fetal stem cells in regeneration of tissues.

## Material and Methods

### Animals

Mice were purchased from the Jackson Laboratory (Bar Harbor, ME). Wild type virgin C57Bl/6 female mice were bred at the animal facility of the University of Southern California, Los Angeles. To investigate the fetal-maternal microchimerism, we crossed wild type virgin C57Bl/6 female mice (6 to 8-week-old) to congenic male mice (6 to 8 weeks-old) carrying an enhanced green fluorescent protein (eGFP) gene. The heterozygous transgenic mice (in C57Bl/6 background) carry an enhanced green fluorescent protein (eGFP) gene which is under the control of a chicken beta-actin promoter and cytomegalovirus enhancer. All the tissues of the eGFP transgenic mice, with the exception of erythrocytes and hair, are green under excitation light. Mice were housed in sterile microisolator cages and fed acidified water and sterilized lab chow. All procedures were performed in accordance with the guidelines of the Animal Care and Use Committee of the University of Southern California.

### Injuries

All injuries were introduced under anesthetization with ketamine (80 mg/Kg). The skin injuries were introduced by shaving the back of the mice and dissecting out epidermis and dermis (approximately 1 cm in diameter). To prevent infection, the wounds were covered with sterile plastic wrap. The spinal cord injuries were introduced by exposing the spine at the T_9_ vertebral level with dorsal incision after shaving the skin.

To prevent miscarriage, we introduced only minor injury to the spinal cord by punching the spine with a 25 gauge needle. The skin wounds were close by a wound clip. Two days after the delivery of the pups, all maternal mice which delivered GFP positive pups were sacrificed. The skins with skin injuries were dissected out. Spines from mice with spinal cord injury were also dissected out and the spinal cords containing the injury site were flushed out with a syringe.

## Results

**Figure 1:**
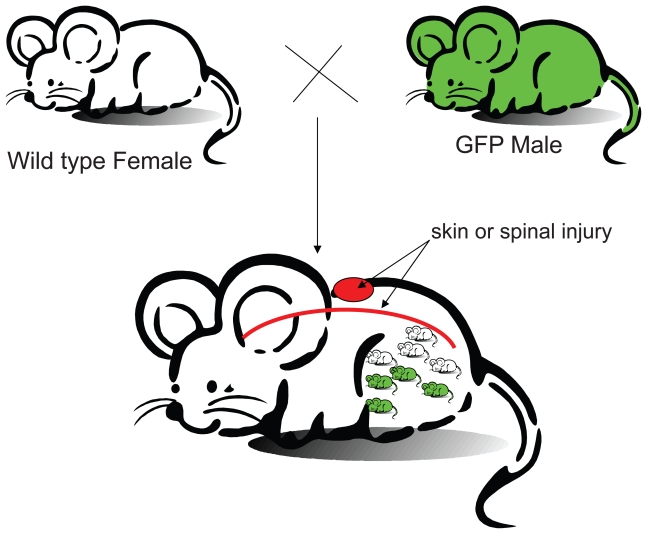
Experimental design for testing the role of injury in microchimerism. Wild type virgin female C57BL/6J mice (8 to 12 weeks old, Jackson Laboratory, ME) were crossed with heterozygous transgenic eGFP male mice. Approximately half of the pups carried the eGFP genes. This is expected for autosomal dominant inheritance of the eGFP gene.

The experimental design is shown at Figure 1. Seven pregnancies were obtained, and all 7 pregnancies resulted in the delivery of 5 to 8 pups from each pregnancy. After the second week of pregnancy, injuries were introduced: skin injuries in 3 mice; spinal cord injuries in 3 mice; one control mouse (which also bred to eGFP males) without injury. New skins with scar tissues were formed in mice with skin injury without infections approximately one week post injury. The injured mice lost the control of their hind legs for 2 to 3 days. After 5 days, all mice with spinal cord injury regained the control of their hind limbs. Minimal injury is introduced here to ensure the successful delivery of the pups. All 3 mice with spinal injury delivered healthy pups in this study. In all deliveries (from all 7 pregnancies), approximately half of the pups are GFP positive and are consistent with the autosomal dominant inheritance of the hemozugous transgenic eGFP gene.

**Figure 2:**
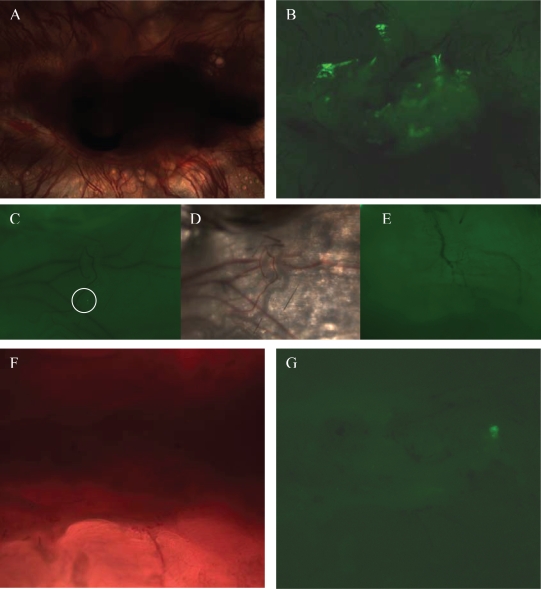
Fetal eGFP cells in injured skin and spinal cord. Both bright field and fluorescent images are taken at 200 × magnification. **A.** scar tissues were formed after skin injury. Massive new blood vessels were visible around the injury site. **B.** Fluorescent image of the scar tissue indicate that patches of fetal eGFP cells are presented close to the blood vessels. **C.** Fluorescent image of the skin next to the skin injury site. There is only one eGFP cells detected (inside the white circle). **D.** The light microscopic image of C. No angiogenesis signs appeared. **E.** Fluorescent image of skin from the control mouse without injury. There is no GFP positive cells detected. **F.** The light microscopic image of a section of the injured spinal cord. **G.** Patches of fetal eGFP cells were shown at the scar tissue around the spinal cord.

It is known that eGFP is not stable in fixed sections. Taking advantage of the thin skin of mice, we took florescent images of the injured area without fixation. Massive new blood vessels were formed around the injury site which indicated the incidence of high angiogenesis events during the recovery of the skin injury (Fig. 2A). Under fluorescent microscope, eGFP cells were observed in the injured sites (Fig. 2B). The eGFP cells were distributed in multiple locations and formed connected GFP positive tissues. There are significant amounts of eGFP cells (approximately 30% of the scar tissue based on fluorescent microscopy) in the scar tissues, and the cells formed integrated GFP positive patches. In contrast, the skin next to the injury site showed no evidence of angiogenesis and only one isolated GFP positive cell was detected (Fig. 2C and 2D). This GFP cell may be a migrating fetal cell. The GFP positive patches are formed by integrated GFP cells, but not a group of single-cells in isolation (Fig. 2B). Skin from control mice showed no incident of angiogenesis and no GFP positive cell was detected (Fig. 2E). These findings agree with other studies which also found that only a few fetal cells were detected in the maternal body.(Khosrotehrani et al. 2005; Tan et al. 2005) We do not find significant amounts of eGFP cells in the injured spinal cords (Fig. 2F and 2G). Only a few eGFP cells were found in the injured sites (Fig. 2G). That result is different from the skin injury result, and may be due to the minimal injury introduced by the needle, and the size of the lesions. The brain, liver and spleen of the mice with skin injury were also examined; no eGFP positive fetal cells were observed under fluorescent microscope. The blood from mice with and without injuries is also examined. GFP positive cells were not identified with fluorescent microscopy.

## Discussion

In this study, we examined the role of fetal cells in maternal body injuries. Fetal-maternal microchimerism has been reported previously.(Liegeois et al. 1981; Philip et al. 1982; Bianchi et al. 1996; Artlett et al. 1998; Bianchi 1998; Krabchi et al. 2001; Khosrotehrani et al. 2003; O’Donoghue et al. 2003; Khosrotehrani et al. 2004; Aractingi et al. 2005; Khosrotehrani et al. 2005; Tan et al. 2005; Khosrotehrani et al. 2006) Fetal cells were found in the maternal body during pregnancy as well as postpartum. However, in these studies only a small amount of fetal cells were found in the maternal body. In our study, we have demonstrated for the first time that injury dramatically increases the amount of fetal cells in the maternal body. The results indicate that injury promoted fetal cells migration toward the injury sites and proliferation at the sites. In contrast, isolated single-cells are detected in areas outside of injury sites. These data suggest that there are few migrating fetal cells in blood and organs, and the integrated GFP positive patches are formed by proliferated and differentiated fetal cells.

It has been reported that stem cells actively migrate to injury site (Park et al. 2006). We also show that fetal cells actively migrate to the maternal injury site. The integrated GFP positive tissues observed in injuries suggest that they are formed by proliferated and differentiated fetal cells rather than migrating fetal cells. The morphology of these GFP cell patches resemble newly formed tissues rather than a group of migrating cells. In addition, the accumulation of migrating fetal cells is most likely to form a continuous GFP positive area rather than separated patches of GFP positive areas (Fig. 2 B). Together with the fact that fetal cells can be detected in the maternal body long after deliveries, these fetal-maternal chimeric cells are likely stem cells. (Bianchi et al. 1996; Khosrotehrani et al. 2005).

Our data also agree with previous reports that loss of a fetus resulted in higher level of fetal microchemirism. (Bianchi et al. 2001; Khosrotehrani et al. 2003) The loss of a fetus could be a result of injury during pregnancy. Fetal cells have also been identified in skin lesions of women with systemic sclerosis, a disease of unknown origin which often occurs in women after their child-bearing years. (Artlett et al. 1998) Different from human studies, our study was conducted with congenic mice which do not have the issue of histocompatibility. This may be the reason that only few fetal cells were identified in women with injury during pregnancy (Artlett et al. 1998).

The fetal-maternal microchimerism is a long lasting event during the development of the fetus. Our data suggest that the level of microchimerism is influenced by whether there is an injury in the maternal body. Whether these fetal-maternal cells could repair the injury of the mother needs to be further investigated by identifying the cell type of the differentiated fetal cells. However, this study suggests that using fetal stem cells for regenerative medicine is possible.
